# Spinal metastases in geriatric patients: a retrospective single-center comparison of mortality and surgical outcomes following neurosurgical treatment

**DOI:** 10.3389/fmed.2026.1897474

**Published:** 2026-07-16

**Authors:** Saif-Eldin Abedellatif, Marija Janjic, Logman Khalafov, Harun Asoglu, Juliane Dittmer, Haitham Alenezi, Ivan Maiseyeu, Mohammed Jaber, Muriel Heimann, Tim Lampmann, Matthias Schneider, Hartmut Vatter, Motaz Hamed, Mohammed Banat

**Affiliations:** Department of Neurosurgery, University Hospital Bonn, Bonn, Germany

**Keywords:** geriatric patients, mortality, neurosurgical procedures, postoperative complications, spinal metastases, survival analysis, treatment outcome

## Abstract

**Background:**

Spinal metastases (SM) are increasingly common. In an aging population, this trend raises concerns regarding the safety and efficacy of surgical treatment (ST) in geriatric patients. This study aimed to compare postoperative outcomes, complications, recurrence, readmission, and mortality between geriatric and non-geriatric patients undergoing ST for SM.

**Methods:**

Our retrospective cohort study included 277 patients who underwent ST for SM between 2012 and 2024. Patients were stratified into non-geriatric (< 70 years, *n* = 161) and geriatric (≥ 70 years, *n* = 116) groups. Clinical and surgical parameters, including complications, recurrence, readmission, and survival, were analyzed.

**Results:**

Geriatric patients had a higher preoperative comorbidity burden and lower functional status. However, early outcomes, such as postoperative complications, readmission rates, and local tumor recurrence, did not differ significantly between age groups. Median overall survival was 12.0 months in patients < 70 years and 10.5 months in patients ≥ 70 years (*p* = 0.026). The multivariable analysis shows that obesity (*p* = 0.028), Surgical stabilization (*p* = 0.043), prolonged postoperative mechanical ventilation (*p* = 0.041), early surgical complications (*p* = 0.009), and a preoperative Karnofsky Performance Scale < 70% (*p* = 0.036) were independently associated with increased 1-year mortality in geriatric patients.

**Conclusion:**

Our data demonstrates that geriatric patients undergoing ST for SM did not experience worse early postoperative outcomes. Careful patient selection based on functional status and perioperative risk factors remains essential.

## Introduction

Spinal metastases represent the most common malignant tumors of the spine and account for up to 70% of all osseous metastases, occurring in approximately 60–70% of patients with systemic cancer during the course of their disease (1). With improving local control of primary tumors and prolonged survival due to advances in systemic therapies and radiotherapy, the number of patients who develop spinal metastases has increased (2). Spinal metastases and metastatic spinal cord compression represent a marker of poor prognosis, with median survival typically 2–3 months once symptomatic cord compression develops (3). Despite modern multimodal treatment concepts, spinal metastases remain associated with considerable morbidity, as pain, neurological deficits, and mechanical instability are common and lead to a marked reduction in quality of life. Surgery is currently a well-known way to treat symptomatic spinal metastases, especially when there is severe neurological impairment, cord compression, or discomfort that does not respond to conventional treatments. Patchell et al. demonstrated that surgical decompression combined with radiotherapy is superior to radiotherapy alone, with 84% of patients maintaining ambulatory capability and having a median survival time of 122 days, compared to 57% and 100 days, respectively (4). The primary goals of neurosurgical intervention are neural decompression, spinal stabilization, and preservation of ambulation to enable ongoing oncological therapy (5). Even in palliative care settings, multiple studies confirm the significant pain alleviation (>50% improvement), neurological rehabilitation (42–64%), and improved functional independence experienced by appropriately selected individuals (6). As life expectancy grows and over 16% of the European population is now 65 years or older, cancer is increasingly prevalent among adults. As the world’s population ages, the prevalence of oncologic diseases is increasingly affecting senior individuals (7). The International Society of Geriatric Oncology (SIOG) advises that 70 years is a pragmatic benchmark for complete geriatric assessment. This advice acknowledges the increasing diversity noted beyond this age, encompassing variations in physiological reserve, frailty, and comorbidity burden (8). Patients aged ≥ 70 years frequently exhibit elevated comorbidity burdens, a frailty prevalence of 25–42%, and a preoperative Karnofsky Performance Scale (KPS) of less than 70%, thereby exacerbating perioperative risks (9, 10). However, recent studies demonstrate comparable neurological recovery rates (41% vs. 44% ambulation enhancement) and 30-day complication rates (27% vs. 23%) in cohorts aged ≥ 70 compared to those aged < 70 after spinal metastases surgery, but with a diminished median survival time (9.8 vs. 14.2 months) (11). Chronological age alone seems to be a less reliable predictor than frailty or performance status. Nonetheless, evidence gaps remain. Previous studies frequently lack age-stratified data on complications, readmissions, recurrence, and 1-year mortality predictions, hence complicating risk–benefit assessments in this diverse population. Therefore, the aim of this retrospective single-center cohort study was to see if risk–benefit assessment could be improved by comparing postoperative outcomes, complications, recurrence, readmission rates, and survival time between geriatric (≥ 70 years) and non-geriatric (< 70 years) patients undergoing neurosurgical treatment for spinal metastases.

## Methods

### Patients and inclusion criteria

This retrospective single-center cohort study identified 300 patients who underwent surgical treatment for spinal metastases between 2015 and 2024. Of these, 277 patients fulfilled the inclusion criteria and were included in the final analysis. The inclusion criteria consisted of histopathologically confirmed spinal metastases at any spinal level, an age of 18 years or older, the availability of complete clinical data, and a minimum follow-up period of 1 year.

### Data recording

Comprehensive clinical and neurological data were collected, including patient age, sex, comorbidities, primary tumor type, spinal metastases location, details of the neurosurgical procedure, extent of vertebral involvement, and preoperative risk status according to the American Society of Anesthesiologists (ASA) classification. Clinical and neurological function was assessed using the American Spinal Injury Association (ASIA) impairment scale. Functional performance on admission was classified as KPS ≥ 70% or KPS < 70%. The Charlson comorbidity index (CCI) was used to quantify the preoperative comorbidity burden. Early postoperative complications were assessed using the publicly available patient safety indicators (PSIs) and hospital-acquired conditions (HACs) measures established by the Agency for Healthcare Research and Quality and the Centers for Medicare and Medicaid Services (12–14). PSIs included events such as myocardial infarction, pressure ulcer, pneumothorax, transfusion reaction, hemorrhage, pulmonary embolism, respiratory failure, deep vein thrombosis, sepsis, wound dehiscence, and accidental puncture or laceration. HACs comprised pneumonia, catheter-associated urinary tract infection, surgical site infection, blood incompatibility, crushing injury, poor glycemic control, fall-related injury, and vascular catheter-associated infection. To identify surgery-specific complications, patient records were additionally reviewed for cerebrospinal fluid leakage, meningitis, implant failure, and new or worsened neurological deficits, collectively defined as spinal surgery-related complications (SSCs). Perioperative complications were defined, in line with previous definitions (15), as any adverse event within 30 days postoperatively, irrespective of the need for reoperation. Overall survival was calculated from the date of spinal metastasis surgery until death or last follow-up. Patients without available follow-up information (e.g., due to continued treatment at external institutions) were excluded from further survival analyses. All analyzed variables were evaluated in relation to overall survival.

### Patient groups

Patients were retrospectively categorized into two groups according to their age at the time of surgery: a geriatric group (≥ 70 years) and a non-geriatric group (< 70 years). This allocation was made in accordance with SIOG’s recommendation, which defines ≥ 70 years as the threshold for comprehensive geriatric assessment.

### Study design

The study adhered to the ethical principles outlined in the 1964 Helsinki Declaration and received approval from the local ethics committee of the University Hospital Bonn (reference no. 067/21). Given the retrospective nature of the study, the acquisition of informed consent from participants was not pursued.

### Exclusion criteria

Patients were excluded if they had spinal metastasis requiring no surgical treatment, if they were under the age of 18 at the time of surgery, or if relevant clinical or follow-up data were missing.

### Statistical analysis and graphical illustrations

Statistical analyses were performed using IBM® SPSS® Statistics for Mac (version 31.0; IBM Corp., Armonk, NY, USA) and RStudio (R version 4.x; RStudio, PBC, Boston, MA, USA). Baseline demographic, tumor-related, surgical, and outcome variables were compared between geriatric (≥ 70 years) and non-geriatric (< 70 years) patients. Categorical variables were analyzed using Pearson’s *χ*^2^ test or Fisher’s exact test, as appropriate. Continuous variables were analyzed using the independent samples *t*-test or Mann–Whitney *U* test, depending on data distribution. Overall survival was estimated using the Kaplan–Meier method and compared between groups using the log-rank test. Kaplan–Meier curves with numbers at risk were generated in R using the survival and survminer packages. To identify independent predictors of mortality, multivariable Cox proportional hazards regression analyses were performed. Separate models were calculated for the entire cohort and for patients aged ≥ 70 years. Results are presented as hazard ratios (HRs) or odds ratios (ORs) with corresponding 95% confidence intervals (CIs). Forest plots were created in R using ggplot2. Sankey diagrams were generated using the R packages ggplot2 and ggalluvial, with data preprocessing performed using readxl, dplyr, and tidyr. All statistical tests were two-sided, and a *p*-value < 0.05 was considered statistically significant.

## Results

### Study population

A total of 277 patients who underwent surgery for spinal metastases were included. Of these, 171 (61.7%) were male and 106 (38.3%) were female. At diagnosis, 161 patients (58.1%) were aged < 70 years and 116 (41.9%) were aged ≥ 70 years. An ASA score >2 was present in 179 patients (64.6%), and 61 patients (22.0%) had a CCI ≥ 10. Polypharmacy (≥ 5 medications) was observed in 117 patients (42.2%). The most common primary tumors were lung (22.7%), prostate (21.3%), and breast (12.6%). The thoracic spine was the most frequently affected region (59.2%). Extracranial metastases were present in 149 patients (53.8%), and spinal cord compression was documented in 244 patients (88.1%). Stabilization procedures were performed in 181 patients (65.3%), while 96 (34.7%) underwent decompression only. Median overall survival was 10.25 months (IQR 3–20), and 1-year mortality was 44.8% (see Table 1 for full details of study population characteristics).

**Table 1:** Patient characteristics (*n* = 277).

Variable	Overall (*n* = 277)
Sex, *n* (%)	
Male/female	171 (61.7)/106 (38.3)
Age at SM diagnosis, *n* (%)	
< 70 years/≥ 70 years	161 (58.1)/116 (41.9)
ASA at SM diagnosis, *n* (%)	
ASA ≤ 2/ASA > 2	98 (35.4)/179 (64.6)
CCI	
< 10/≥ 10	216 (78.0)/61 (22.0)
Polypharmacy, *n* (%)	
No	109 (39.4)
Yes (≥ 5)	117 (42.2)
Excessive (≥ 10)	49 (17.7)
Missing	2 (0.7)
Preoperative ATM, *n* (%)	
Antiplatelet agents	25 (9.0)
DOACs	13 (4.7)
VKAs	0 (0.0)
Heparin	49 (17.7)
Combination	5 (1.8)
No	183 (66.1)
Missing	2 (0.7)
Primary tumor, *n* (%)	
Lung cancer	63 (22.7)
Breast cancer	35 (12.6)
Prostate cancer	59 (21.3)
Plasmacytoma	13 (4.7)
GI cancer	29 (10.5)
Kidney cancer	24 (8.7)
Other pathologies	44 (15.9)
CUP	10 (3.6)
First manifestation as SM, *n* (%)	
Synchronous	114 (41.2)
Metachronous	151 (54.5)
Missing	12 (4.3)
TTP, PT to SM diagnosis, *n* (%)	
< 1 year	119 (43.0)
≥ 1 year, < 3 years	62 (22.4)
≥ 3 years, < 5 years	20 (7.2)
≥ 5 years	61 (22.0)
Missing	15 (5.4)
Previous treatment, *n* (%)	
Yes/No	144 (52.0)/133 (48.0)
SM locations, *n* (%)	
Craniocervical	2 (0.7)
Cervical vertebrae	26 (9.4)
Cervicothoracic	20 (7.2)
Thoracic vertebrae	164 (59.2)
Thoracolumbar	17 (6.1)
Lumbar spine	41 (14.8)
Lumbosacral	7 (2.5)
Spinal cord compression, *n* (%)	
Yes/no	244 (88.1)/33 (11.9)
Involved segments, *n* (%)	
≤ 2/≥ 3 segments	165 (59.6)/112 (40.4)
Extraspinal metastases, *n* (%)	
Yes/No	149 (53.8)/126 (45.5)
Extracranial metastases sites, *n*	
Median (IQR)/range	1 (0–2)/7
Surgery, *n* (%)	
Only decompression	96 (34.7)
Stabilization	181 (65.3)
Early postoperative complications, *n* (%)	
PSIs	
Yes/no	141 (50.9)/136 (49.1)
HACs	
Yes/No	87 (31.4)/190 (68.6)
Specific SSCs	
Yes/no	40 (14.4)/237 (85.6)
Operative revision, *n* (%)	
Yes/No	26 (9.4)/251 (90.6)
LOS, days	
Median (IQR)/range	12 (7–18)/88
Preoperative ASIA, *n* (%)	
Good (D, E)/poor (A, B, C)	190 (68.6)/87 (31.4)
Preoperative KPS, *n* (%)	
≥ 70%/< 70	169 (61.0)/105 (37.9)
Postoperative ASIA, *n* (%)	
Good (D, E)/poor (A, B, C)	192 (69.3)/85 (30.7)
Postoperative KPS, *n* (%)	
≥ 70%/< 70	177 (63.9)/100 (36.1)
Postoperative ST, (%)	
Yes	170 (61.4)
No	89 (32.1)
Missing	18 (6.5)
Postoperative RXT, *n* (%)	
Yes	169. (61.0)
No	90 (32.5)
Missing	18 (6.5)
Readmission in 30 days, *n* (%)	
Yes	14 (5.1)
No	241 (87.0)
Missing	22 (7.9)
Readmission in 3 months, *n* (%)	
Yes	21 (7.6)
No	225 (81.2)
Missing	31 (11.2)
Overall survival, months	
Median (IQR)/range	10.25 (3–20)/2,238
Missing	19
One-year mortality, *n* (%)	
Yes	124 (44.8)
No	121 (43.7)
Missing	32 (11.6)

ASA, American Society of Anesthesiology; ASIA, American Spinal Injury Association; ATM, antithrombotic medication; CCL, clinical comorbidity level; CUP, cancer of unknown primary; DOACs, direct oral anticoagulants; GI, gastrointestinal; HAC, hospital-acquired condition; IQR, interquartile range; KPS, Karnofsky Performance Scale; LOS, length of stay; PSI, patient safety indicator; PT, primary tumor; RXT, radiotherapy; SM, spinal metastases; SSC, spinal surgery-related complication; ST, systemic therapy; TTP, time to progression; VKA, vitamin K antagonist.

### Baseline characteristics and preoperative status according to patient age

Geriatric patients (≥ 70 years) more often had an ASA score >2 than younger patients (74.1% vs. 57.8%, *p* = 0.005). They were also more frequently treated with antithrombotic agents (40.5% vs. 28.0%, *p* = 0.013). No significant differences between age groups were detected for sex, CCI, BMI, or polypharmacy (all *p* > 0.05; Table 2A). Prostate cancer was significantly more frequent in geriatric patients than in younger patients (31.9% vs. 13.7%, *p* = 0.001), whereas lung and gastrointestinal cancers were more prevalent in the younger group (lung: < 70 26.7% vs. ≥ 70 17.2%, *p* = 0.049; gastrointestinal: < 70 14.2% vs. ≥ 70 5.2%, *p* = 0.031). No significant differences between age groups were observed regarding timing of metastasis, tumor location, spinal cord compression, or spinal instability (Table 2B). The younger group had significantly more extraspinal metastases than the geriatric group (median 1 vs. 0, *p* = 0.003), with a higher frequency of bone (47.5% vs. 35.7%, *p* = 0.05) and liver metastases (16.9% vs. 7.8%, *p* = 0.028) (Table 2C). Preoperative functional status was significantly impaired in geriatric patients, with a lower proportion of patients presenting with KPS ≥ 70% (51.3% vs. 69.2%, *p* = 0.003). Preoperative ASIA grade did not differ significantly between groups (*p* = 0.231) (Table 2D).

**Table 2:** Characteristics of surgically treated patients with spinal metastasis, stratified by age (≥ 70 vs. < 70 years).

Variable	< 70 years (*n* = 161)	≥ 70 years, (*n* = 116)	*p*-value
A) Patient demographics			
Sex, *n* (%)			0.109
Male/female	93 (57.8)/68 (42.2)	78 (67.2)/38 (32.8)	
ASA at SM diagnosis, *n* (%)			**0.005**
ASA ≤ 2/ASA > 2	68 (42.2)/93 (57.8)	30 (25.9)/86 (74.1)	
BMI, *n* (%)			0.818
Underweight/normal	3 (1.9)/64 (39.8)	2 (1.7)/41 (35.3)	
Overweight/obesity	78 (48.4)/16 (9.9)	63 (54.3)/10 (8.3)	
CCI, *n* (%)			0.557
< 10/≥ 10	128 (79.5)/33 (20.5)	88 (75.9)/28 (24.1)	
Polypharmacy, *n* (%)			0.492
No/yes (≥ 5)/excessive (≥ 10)	65 (40.4)/71 (44.1)/25 (15.5)	44 (38.6)/46 (40.4)/24 (21.1)	
Preoperative ATM, *n* (%)			**0.013**
No/yes	116 (72)/45 (27.95)	67 (58.8)/47 (40.51)	
Antiplatelet agents	10 (6.2)	15 (13.2)	
DOACs	4 (2.5)	9 (7.9)	
VKAs	0 (0)	0 (0)	
Heparin	30 (18.6)	19 (16.7)	
Combination	1 (0.6)	4 (3.5)	
B) Tumor characteristics			
Primary tumor, *n* (%)			
Lung cancer	43 (26.7)	20 (17.2)	**0.049**
Breast cancer	22 (13.7)	13 (11.2)	0.544
Prostate cancer	22 (13.7)	37 (31.9)	**0.001**
Plasmacytoma	7 (4.3)	6 (5.2)	
GI cancer	23 (14.3)	6 (5.2)	**0.031**
Kidney cancer	14 (8.7)	10 (8.6)	
Other pathologies	26 (16.1)	18 (15.5)	
CUP	4 (2.5)	6 (5.2)	
TTP, PT to SM diagnosis, *n* (%)			0.230
< 1 year	64 (41.8)	55 (50.5)	
≥ 1 year, < 3 years	43 (28.1)	19 (17.4)	
≥ 3 years, < 5 years	12 (7.8)	8 (7.3)	
≥ 5 years	34 (22.2)	27 (24.8)	0.123
First manifestation as SM, *n* (%)			
Synchronous/metachronous	61 (39.1)/95 (60.9)	53 (48.6)/56 (51.4)	
Previous treatment, *n* (%)			0.574
Yes/no	86 (53.4)/75 (46.6)	58 (50) /58 (50)	
SM locations, *n* (%)			0.501
Craniocervical	1 (0.6)	1 (0.9)	
Cervical vertebrae	19 (11.8)	7 (6)	
Cervicothoracic	13 (8.1)	7 (6)	
Thoracic vertebrae	88 (54.7)	76 (65.5)	
Thoracolumbar	9 (5.6)	8 (6.9)	
Lumbar spine	26 (16.1)	15 (12.9)	
Lumbosacral	5 (3.1)	2 (1.7)	
Involved segments, *n* (%)			0.785
≤ 2 segments/≥ 3 segments	97 (60.2)/64 (39.8)	68 (58.6)/48 (41.4)	
Spinal cord compression, *n* (%)			0,758
Yes/no	141 (87.6)/20 (12.4)	103 (88.8)/13 (11.2)	
Spinal instability, *n* (%)			1
Yes/no	104 (66.7)/52 (33.3)	74 (66.7)/37 (33.3)	
C) Systemic disease burden			
Extraspinal metastases, *n* (%)			0.073
Yes/no	94 (58.8)/66 (41.3)	55 (47.8)/60 (52.2)	
Numbers of metastases sites			**0.003**
Median (IQR)/range	1 (0–2)/7	0 (0–1)/6	
Localization of extracranial metastases, *n* (%)			
Lymph node	43 (26.9)	21 (18.3)	0.095
Bone	76 (47.5)	41 (35.7)	**0.05**
Liver	27 (16.9)	9 (7.8)	**0.028**
Lung	25 (15.6)	14 (12.2)	0.418
Subcutaneous	3 (1.9)	1 (0.9)	0.642
Stomach	1 (0.6)	0 (0)	1
Small intestine	2 (1.3)	0 (0)	0.512
Colorectal	1 (0.6)	0 (0)	1
Renal	1 (0.6)	0 (0)	1
Adrenal	11 (6.9)	7 (6.1)	0.794
Pancreatic	6 (3.8)	2 (1.7)	0.475
Splenic	2 (1.3)	0 (0)	0.512
Brain	14 (8.8)	5 (4.3)	0.156
Others	15 (9.5)	2 (1.7)	**0.009**
D) Preoperative functional and neurological status			
Preoperative ASIA, *n* (%)			0.231
Good (D, E)/poor (A, B, C)	115 (71.4)/46 (28.6)	75 (64.7) /41 (35.3)	
Preoperative KPS, *n* (%)			**0.003**
≥ 70%/< 70	110 (69.2)/49 (30.8)	59 (51.3)/56 (48.7)	
E) Surgical treatment and oncologic therapy			
Surgery, *n* (%)			0.573
Only decompression/stabilization	58 (36)/103 (64)	38 (32.8)/78 (67.2)	
Operative time, minutes			0.92
Median (IQR)/range	185 (132–256)/580	182 (132–269)/562	
Intraoperative blood loss, mL			0.622
Median (IQR)/range	600 (300–1,100)/11,450	600 (350–1,150)/6,950	
Perioperative blood transfusion, *n* (%)			0.738
Yes/no	44 (37.3)/74 (62.7)	36 (39.6)/55 (60.4)	
Postoperative ST, *n* (%)			0.616
Yes/No	101 (66.9)/50 (33.1)	69 (63.9)/39 (36.1)	
Postoperative RXT, *n* (%)			0.237
Yes/No	103 (68.2)/48 (31.8)	66 (61.1)/42 (38.9)	
F) Postoperative outcomes			
Postoperative blood transfusion, *n* (%)			0.494
Yes/no	4 (2.5)/153 (97.5)	5 (4.5)/105 (95.5)	
Postoperative PMV, *n* (%)			0.929
No/yes	143 (89.9)/16 (10.1)	103 (90.4)/11 (9.6)	
Duration 24 h/48 h/72 h	14 (8.8)7/7 (4.4)/4 (2.5)	10 (8.8)/6 (5.3)/4 (3.5)	0.166
Length of stay, days			
Median (IQR)/range	16 (11–35.5)/59	43 (28–66.5)/77	0.610
ICU-LOS, days			
Median (IQR)/range	1 (0–6)/20	5 (3–6.5)/7	0.638
ICU proportion of LOS, (%)			
Median (IQR)/range	6.006 (0–13.594)/57.69	7.6923 (6.6239–13.1485)/13.05	
Early postoperative complications, *n* (%)			0.371
PSIs	78 (48.4)	63 (54.3)	**0.005**
HACs	40 (24.8)	40 (40.5)	0.355
Specific SSCs	26 (16.1)	14 (12.1)	0.236
Postoperative revision, *n* (%)			
Yes/no	18 (11.2)/143 (88.8)	8 (6.9)/108 (93.1)	0.801
Time to revision, days			
Median (IQR)/range	35.5 (17–56)/720	26 (13.5–55.5)/84	0.289
Postoperative ASIA, *n* (%)			
Good (D, E)/Poor (A, B, C)	116 (72)/45 (28)	76 (65.5)/40 (34.5)	**0.049**
Postoperative KPS, *n* (%)			
≥ 70%/< 70	111 (68.9)/50 (31.1)	66 (59.9)/50 (43.1)	0.892
Local tumor recurrence, *n* (%)			
Yes/no	24 (16.3)/123 (83.7)	16 (15.7)/86 (84.3)	0.384
Recurrence free interval, months			
Median (IQR)	2.5 (1.5–10)	3 (2–7)	
Range	30	10	0.107
Readmission in 30 days, *n* (%)			
Yes/no	11 (7.5)/136 (92.5)	3 (2.8)/105 (97.2)	1
Readmission in 3 months, *n* (%)			
Yes/no	12 (8.6)/128 (91.4)	9 (8.5)/97 (91.5)	0.625
One year mortality, *n* (%)			
Yes/no	71 (49.3)/73 (50.7)	53 (52.5)/48 (47.5)	

ASA, American Society of Anesthesiology; ASIA, American Spinal Injury Association; ATM, antithrombotic medication; BMI, body mass index; CCL, clinical comorbidity level; CUP, cancer of unknown primary; DOACs, direct oral anticoagulants; GI, gastrointestinal; HACs, hospital-acquired conditions; ICU, intensive care unit; IQR, interquartile range; KPS, Karnofsky Performance Scale; LOS, length of stay; PMV, postoperative mechanical ventilation; PSIs, patient safety indicators; PT, primary tumor; RXT, radiotherapy; SM, spinal metastases; SSCs, spinal surgery-related complications; ST, systemic therapy; TTP, time to progression; VKAs, vitamin K antagonists.Bold values indicate statistically significant differences (*p* < 0.05).

### Surgical and postoperative outcomes according to patient age

**Figure 1:**
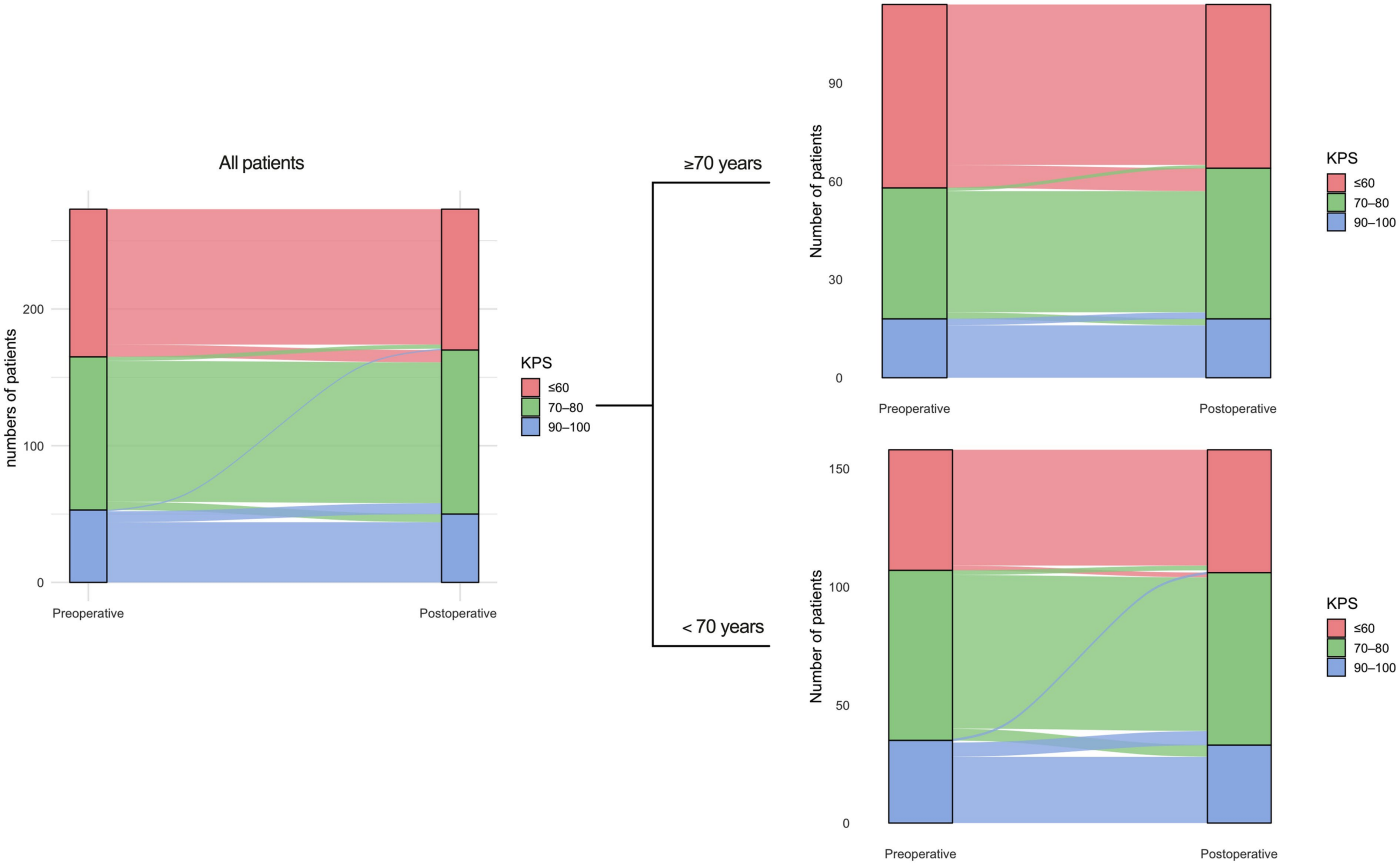
Sankey diagram showing pre- and postoperative Karnofsky Performance Scale (KPS) in the total cohort and by age (< 70 vs. ≥ 70 years). Patients were grouped into three KPS categories (≤60, 70–80, 90–100). The flow width reflects the number of patients changing categories. Most patients remained in their preoperative KPS category, with only minor postoperative shifts in both age groups.

Our analysis showed no significant differences between age groups regarding surgical approach, operative time, intraoperative blood loss, or perioperative transfusion rates (all *p* > 0.05; Table 2E). Hospital-acquired complications were more frequent in geriatric patients (40.5% vs. 24.8%, *p* = 0.005), whereas PSIs and specific surgical complications did not differ significantly. Postoperative ASIA and KPS scores in the geriatric group did not worsen compared with preoperative values. Although improvements were observed, they were not statistically significant (postoperative KPS > 70 59.9% vs. preoperative KPS < 70 51.3%, *p* = 0.109; postoperative ASIA “good” 64.7% vs. preoperative ASIA “good” 65.5%, *p* = 1; Figure 1 and Table 2D,F). Length of stay, ICU stay, and readmission rates at 30 days and 3 months did not differ significantly between age groups (Table 2F).

### Overall survival after surgical treatment of spinal metastases stratified by age

**Figure 2:**
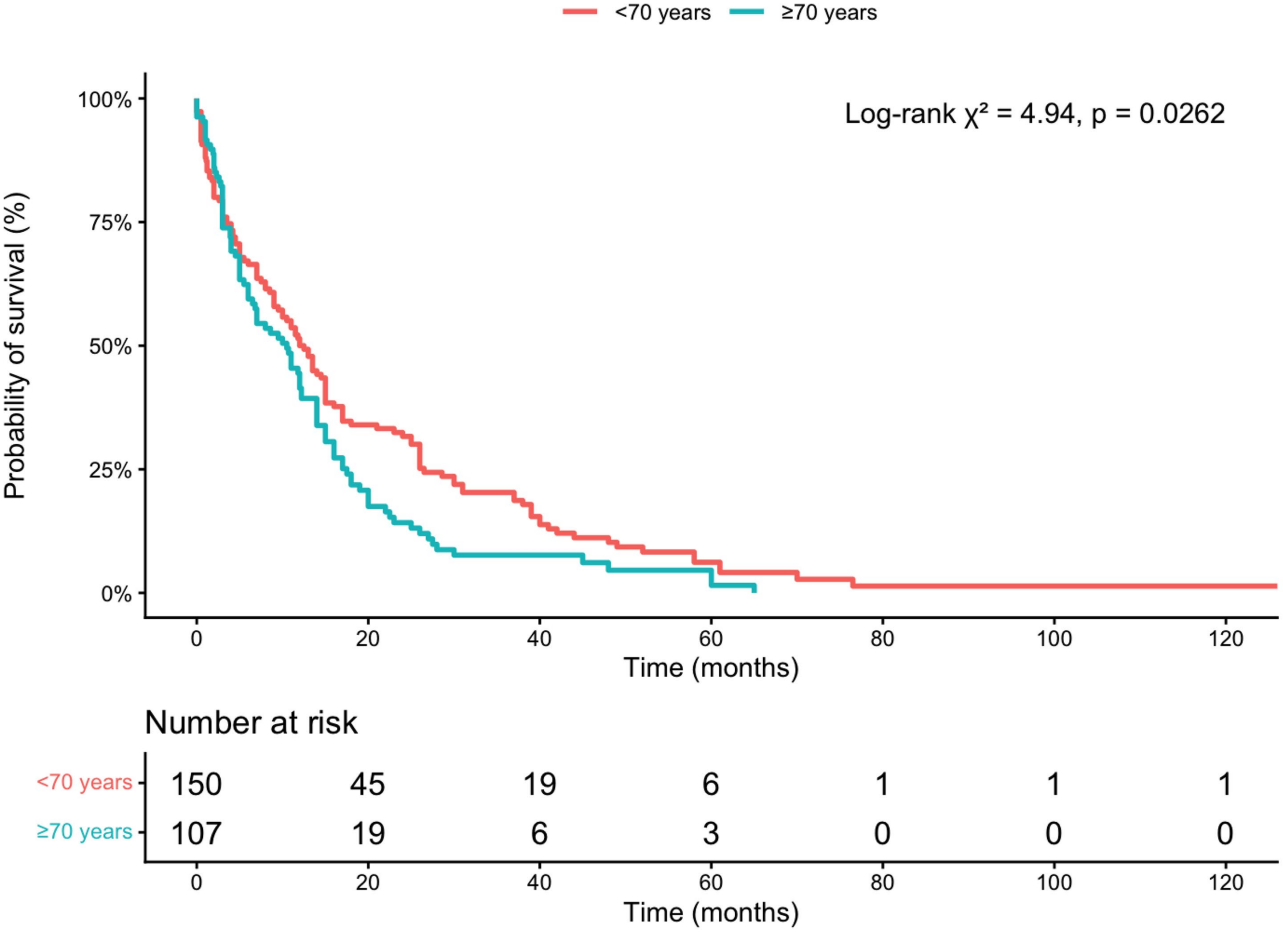
Kaplan–Meier survival analysis stratified by age (<70 vs. ≥70 years).

Median overall survival after surgery for spinal metastases was 12.0 months (95% CI 9.58–14.42) in patients aged < 70 years and 10.5 months (95% CI 6.81–14.19) in those aged ≥ 70 years. Kaplan–Meier analysis showed a significant survival difference between age groups (log-rank *χ*^2^ = 4.942, *p* = 0.026; Figure 2). One-year mortality was 49.3% in patients < 70 years and 52.5% in patients ≥ 70 years, a difference that did not reach statistical significance (*p* = 0.625; Table 2F).

### Multivariable predictors of 1-year mortality after surgical treatment of spinal metastases in overall and geriatric cohorts

**Figure 3:**
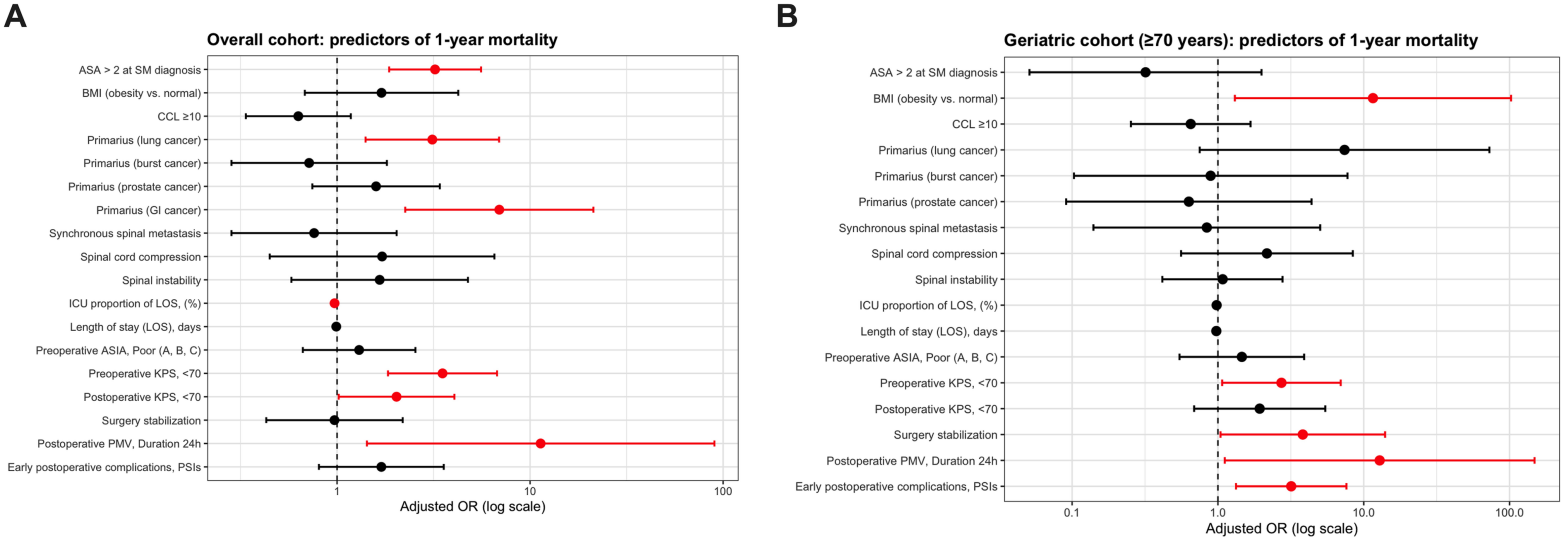
Multivariable predictors of 1-year mortality after surgery for spinal metastases. Forest plots illustrate adjusted odds ratios and 95% confidence intervals from multivariable logistic regression analyses in the overall cohort **(A)** and in patients aged ≥ 70 years **(B)**. The dashed vertical line indicates the null effect (OR = 1). Statistically significant predictors (*p* < 0.05) are shown in red. ASA, American Society of Anesthesiology; ASIA, American Spinal Injury Association; BMI, body mass index; CCL, clinical comorbidity level; CI, confidence interval; GI, gastrointestinal; ICU, intensive care unit; KPS, Karnofsky Performance Scale; LOS, length of stay; OR, odds ratio; PMV, postoperative mechanical ventilation; PSIs, patient safety indicators; SM, spinal metastases.

In the overall cohort, multivariable logistic regression analysis identified several independent predictors of 1-year mortality (Figure 3A; full model shown in Supplementary Table S1). An ASA score >2 was significantly associated with increased mortality (OR 3.22, 95% CI 1.86–5.57, *p* = 0.001). Primary lung cancer (OR 3.12, 95% CI 1.41–6.91, *p* = 0.005) and gastrointestinal cancer (OR 6.93, 95% CI 2.26–21.29, *p* = 0.001) were independently associated with higher 1-year mortality. Functional status was strongly associated with outcome: preoperative KPS < 70% (OR 3.52, 95% CI 1.84–6.75, *p* = 0.001) and postoperative KPS < 70% (OR 2.03, 95% CI 1.02–4.06, *p* = 0.044) were significant predictors. Prolonged postoperative mechanical ventilation (≥ 24 h) was associated with markedly increased mortality risk (OR 11.35, 95% CI 1.43–90.14, *p* = 0.022). Additionally, a higher proportion of days in ICU relative to total hospital stay was independently associated with mortality (OR 0.97, 95% CI 0.94–0.998, *p* = 0.037). Other variables, including BMI, CCI ≥ 10, spinal cord compression, spinal instability, synchronous metastasis, and surgical stabilization, were not independently associated with 1-year mortality (Supplementary Table S1).

In the geriatric cohort (≥ 70 years), multivariable analysis revealed a distinct pattern of predictors (Figure 3B; full model shown in Supplementary Table S2). Obesity was independently associated with increased 1-year mortality (OR 11.57, 95% CI 1.31–102.45, *p* = 0.028). Surgical stabilization (OR 3.82, 95% CI 1.04–14.01, *p* = 0.043) and prolonged postoperative mechanical ventilation (OR 12.87, 95% CI 1.12–148.45, *p* = 0.041) were also significant predictors. Early postoperative PSI events were independently associated with mortality (OR 3.18, 95% CI 1.33–7.61, *p* = 0.009). Preoperative KPS < 70% remained an independent predictor in geriatric patients (OR 2.73, 95% CI 1.07–6.96, *p* = 0.036), whereas tumor-related variables and comorbidity burden did not show statistically significant associations in this subgroup (Supplementary Table S2).

## Discussion

The care and treatment of older patients represent an increasing challenge across several fields of medicine. In everyday clinical practice, these considerations, encompassing not only medical but also socioeconomic and ethical aspects, are central to patient care. As the population ages, these issues are becoming increasingly prominent (2, 16). Our research group at the university center focuses on clinical parameters that may influence treatment decisions and outcomes in geriatric patients (17).

Spinal metastases represent a manifestation of advanced systemic malignancy and cause significant morbidity in affected patients (18). If factors are present that may influence the overall treatment approach, whether favorably or unfavorably, they should be incorporated into the management of spinal metastases (19, 20).

The aim of the current study was to determine whether geriatric patients (≥ 70 years) with spinal metastases were at greater risk following surgical treatment than younger populations, and to identify which factors might positively or negatively influence the postoperative course.

In our cohort, the underlying primary tumor differed significantly between age groups. Prostate cancer was more frequent in geriatric patients, whereas lung and gastrointestinal cancers were more common in younger patients. In addition, younger patients presented with a greater systemic metastatic burden, as reflected by a higher number of extraspinal metastases and more frequent bone and liver involvement. This age-dependent tumor distribution is in line with previous reports showing that the spectrum of spinal metastases varies according to primary tumor type and patient age, with prostate cancer being more common in older men and lung or gastrointestinal primaries contributing substantially to metastatic spinal disease in broader cohorts (21–23). In our cohort older age did not play a significant role in the decision to operate; nor, compared with younger patients, did the older patient population exhibit a significantly increased risk of postoperative complications. A literature review shows that few studies address the topic of our research group. Many studies focus on spinal care for conditions other than spinal metastases, such as degenerative diseases and traumatic spinal injuries (24, 25). This is particularly relevant because, despite a significantly worse preoperative functional status in geriatric patients, with a lower proportion of patients achieving KPS ≥ 70%, the preoperative ASIA grade did not differ significantly between the two age groups. Notably, postoperative functional status improved in the geriatric cohort, with a reduction in the proportion of patients with KPS < 70%, suggesting that surgery may provide meaningful functional benefit in carefully selected older patients. These findings support the growing view that chronological age alone should not be used to deny surgical treatment when functional reserve and neurological status remain acceptable (26). Geriatric patients showed no significant increase in postoperative complications or perioperative morbidity, consistent with studies on spinal stabilization/decompression across various conditions (27, 28). Risks were primarily linked to comorbidities, ASA score, and BMI rather than tumor-specific factors—patterns observed in other geriatric surgical populations (29–31). In a retrospective cohort study that included 1,796 patients with spinal metastases, Elsamadicy et al. showed that mortality, extended length of stay, and postoperative complications were independent predictors of non-routine discharge. Our data show slightly lower rates across all aspects than this study; this may be because our cohort was somewhat smaller (32). Survival analysis showed a significant difference between age groups in our study, but the absolute difference in median overall survival was small. This suggests that age alone has limited prognostic value, and that survival after surgery for spinal metastases is more strongly influenced by tumor biology and perioperative factors than by chronological age. An increasingly important aspect of the management of spinal metastases in geriatric patients is interdisciplinary collaboration among various medical departments. This approach is practiced and implemented at our hospital, as documented in numerous scientific publications (33, 34). A more detailed understanding of the nature of the primary tumor, as well as knowledge of molecular genetics, is associated with better outcomes. Therefore, some authors call for a more detailed investigation of the nature of the tumor causing spinal metastases (35). In our cohort, gastrointestinal and bronchial tumors were associated with higher mortality rates. The preoperative neurological status according to KPI also plays a significant role in the treatment of spinal metastases and influences the outcome (36). Consistent with this concept, our multivariable analysis confirmed that mortality was driven by perioperative and disease-related factors rather than age. Key predictors included ASA score, lung/gastrointestinal primaries, impaired KPS, and prolonged ventilation. In geriatric patients, obesity, surgical stabilization, PSI events, and preoperative KPS < 70% predominated. These findings support age-inclusive surgical decision-making based on comprehensive risk assessment rather than chronological age alone.

This study demonstrated that geriatric patients with spinal metastases did not face significantly higher complication rates or early adverse outcomes from surgery than younger patients. Age alone should not preclude intervention; a comprehensive assessment of tumor biology, functional status, and perioperative risks is a better guide for decision-making.

## Conclusion

In this study, geriatric patients (≥ 70 years) undergoing surgery for spinal metastases had higher overall mortality than younger patients, but complication rates and functional outcomes were comparable between age groups. Notably, age itself was not an independent predictor of 1-year mortality. These findings suggest that chronological age alone should not preclude surgical treatment. Decisions should instead be based on the patient’s overall condition, tumor characteristics, and functional status.

### Limitations

The present study has several limitations. Patients were not randomized, and the decision for surgical treatment was made by the treating neurosurgeon based on individual clinical judgment. The acquisition of data was retrospective. Therefore, our findings are subject to well-known and well-described sources of bias, particularly selection bias, as patients deemed suitable for surgery may have represented a preselected subgroup in both the geriatric and younger cohorts. Furthermore, the comparison between geriatric and younger patients was based primarily on chronological age. However, chronological age alone may not adequately reflect the biological and functional condition of the patient. Important factors such as frailty status, preoperative functional reserve, comorbidity burden, nutritional status, and overall performance status were not consistently available and it was therefore not possible to fully integrate them into the analysis. These variables may have substantially influenced postoperative complications, recovery, and survival, and may have differed considerably within the geriatric cohort itself.

In addition, the study population comprised a heterogeneous group of primary tumor entities and varying extents of metastatic disease. Since tumor biology, systemic disease burden, and prognosis may differ significantly between younger and older patients, this heterogeneity may have influenced the comparison between the two age groups and may limit the generalizability of the findings. Moreover, detailed oncological follow-up data, particularly regarding adjuvant treatments such as chemotherapy, radiotherapy, immunotherapy, or targeted systemic therapies, were not comprehensively available. These therapies may have substantially affected postoperative outcome and overall survival, so differences in treatment patterns between geriatric and younger patients could not be fully assessed. Finally, the present study was conducted at a single center, which may limit external validity. Prospective multicenter studies with larger and more homogeneous patient populations are warranted to validate these findings and to better define the impact of age-related factors on surgical outcomes in patients with spinal metastases.

## Data Availability

The original contributions presented in the study are included in the article/Supplementary material, further inquiries can be directed to the corresponding author.

## Glossary

ASA: American Society of Anesthesiology
ASIA: American Spinal Injury Association
ATM: Antithrombotic medication
BMI: Body mass index
CCI: Charlson comorbidity index
CI: Confidence interval
CUP: Cancer of unknown primary
DOAC: Direct oral anticoagulant
GI: Gastrointestinal
HAC: Hospital-acquired condition
HR: Hazard ratio
ICU: Intensive care unit
IQR: Interquartile range
KPS: Karnofsky Performance Scale
LOS: Length of stay
N: Numbers
OR: Odds ratio
PMV: Postoperative mechanical ventilation
PSI: Patient safety indicator
PT: Primary tumor
RXT: Radiotherapy
SIOG: International Society of Geriatric Oncology
SM: Spinal metastasis
SSC: Spinal surgery-related complication
ST: Systemic therapy
TTP: Time to progression
VKA: Vitamin K antagonist
