# Supplementary material for: Spinal metastases in geriatric patients: a retrospective single-center comparison of mortality and surgical outcomes following neurosurgical treatment
Source: Front Med (Lausanne). 2026 Jul 16;13:1897474. doi: 10.3389/fmed.2026.1897474 (PMC13422460; doi:10.3389/fmed.2026.1897474)
Supplement: Supplementary file 1 [file Table_1.docx]

**Supplementary materials**

**Tables**

**Table S1.** Multivariable regression analysis for predictors of 1-year mortality in all patients with spinal metastases

| Factors | Adjusted OR | 95% CI | *p*-value |
| --- | --- | --- | --- |
| 1. **Patient demographics**   Female sex  **ASA at SM diagnosis, score**  **ASA > 2 at SM diagnosis**  BMI (obesity vs. normal)  CCI ≥ 10  **Polypharmacy (none vs. excessive (≥ 10))**  Preoperative antithrombotic medications   1. **Tumor characteristics**   **Primary tumor (lung cancer)**  Primary tumor (breast cancer)  Primary tumor (prostate cancer)  **Primary tumor (GI cancer)**  Primary tumor (kidney cancer)  Ki-67, index  Synchronous spinal metastasis  TTP, PT to SM diagnosis, years  **Previous treatment**  Thoracic vertebrae location  Involved segments, numbers  Spinal cord compression  Spinal instability   1. **Systemic disease burden**   Extraspinal metastases  Metastases sites, numbers   1. **Surgical and postoperative treatment**   Surgery stabilization  **Operative time, minutes**  Intraoperative blood loss, mL  Perioperative blood transfusion  Postoperative systematic therapy  Postoperative RXT   1. **Functional and neurological status before & after surgery**   Preoperative ASIA, poor (A, B, C)  **Preoperative KPS, < 70**  Postoperative ASIA, poor (A, B, C)  **Postoperative KPS, < 70**   1. **Postoperative outcomes**   **PMV, duration 24h**  Length of stay, days  **ICU proportion of LOS, %**  Early postoperative complications, PSIs  Early postoperative complications, HACs  Early postoperative complications, specific SSCs  Postoperative revision  Time to revision, days  Local tumor recurrence  Readmission in 30 days  Readmission in 3 months | 0.614  0.349  3.22  1.7  0.63  0.291  0.98  3.117  0.717  1.593  6.93  1.396  0.993  0.761  1.068  3.176  1.98  0.872  1.712  1.663  1.333  0.945  0.97  1.005  1  1.427  0.237  0.978  1.302  3.52  1.955  2.034  11.346  0.991  0.971  1.697  1.324  0.654  0.243  1.007  0.785  0.779  1.119 | 0.363 – 1.037  0.228 – 0.535  1.863 – 5.572  0.681 – 4.247  0.337 – 1.179  0.127 – 0.666  0.532 – 1.804  1.405 – 6.914  0.284 – 1.813  0.745 – 3.408  2.256 – 21.29  0.526 – 3.706  0.972 – 1.015  0.284 – 2.037  0.983 – 1.16  1.25 – 8.074  0.901 – 4.349  0.635 – 1.197  0.448 – 6.536  0.58 – 4.769  0.621 – 2.865  0.727 – 1.23  0.43 – 2.19  1.001 – 1.01  1 – 1  0.596 – 3.419  0.042 – 1.355  0.172 – 5.549  0.665 – 2.548  1.837 – 6.746  0.947 – 4.036  1.02 – 4.056  1.428 – 90.138  0.964 – 1.019  0.944 – 0.998  0.806 –3.572  0.623 – 2.815  0.256 – 1.672  0.032 – 1.878  0.985 – 1.030  0.387 – 1.594  0.233 – 2.608  0.418 – 2.997 | 0.068  **0.001**  **0.001**  0.256  0.149  **0.003**  0.947  **0.005**  0.483  0.230  **0.001**  0.503  0.548  0.586  0.122  **0.015**  0.089  0.396  0.432  0.344  0.461  0.676  0.941  **0.022**  0.747  0.425  0.106  0.978  0.441  **0.001**  0.07  **0.044**  **0.022**  0.522  **0.037**  0.164  0.466  0.375  0.175  0.519  0.503  0.686  0.822 |

ASA, American Society of Anesthesiology; ASIA, American Spinal Injury Association; BMI, body mass index; CCI, Charlson comorbidity index; CI, confidence interval; GI, gastrointestinal; HACs, hospital-acquired conditions; ICU, intensive care unit; KPS, Karnofsky Performance Scale; LOS, length of stay; OR, odds ratio; PMV, postoperative mechanical ventilation; PT, primary tumor; PSIs, patient safety indicators; RXT, radiotherapy; SM, spinal metastases; SSCs, spinal surgery-related complications; TTP, time to progression.

**Table S2.** Multivariable regression analysis for predictors of 1-year mortality in patients with spinal metastases aged ≥ 70 years

| Factors | Adjusted OR | 95% CI | *p*-value |
| --- | --- | --- | --- |
| 1. **Patient demographics**   Female sex  **ASA at SM diagnosis, score**  ASA > 2 at SM diagnosis  **BMI (obesity vs. normal)**  CCI ≥ 10  Polypharmacy (none vs. excessive (≥ 10))  Preoperative antithrombotic medications   1. **Tumor characteristics**   Primary tumor (lung cancer)  Primary tumor (breast cancer)  Primary tumor (prostate cancer)  Primary tumor (kidney cancer)  Ki-67, index  Synchronous spinal metastasis  TTP, PT to SM diagnosis, years  Previous treatment  Thoracic vertebrae location  Involved segments, numbers  Spinal cord compression  Spinal instability   1. **Systemic disease burden**   Extraspinal metastases  Metastases sites, numbers   1. **Surgical treatment**   **Surgery stabilization**  Operative time, minutes  Intraoperative blood loss, mL  Perioperative blood transfusion   1. **Functional and neurological status before & after surgery**   Preoperative ASIA, poor (A, B, C)  **Preoperative KPS, < 70**  Postoperative ASIA, poor (A, B, C)  Postoperative KPS, < 70   1. **Postoperative outcomes**   **PMV, duration 24h**  Length of stay, days  ICU proportion of LOS, **(%)**  **Early postoperative complications, PSIs**  Early postoperative complications, HACs  Early postoperative complications, specific SSCs  Time to revision, days  Local tumor recurrence  Readmission in 30 days  Readmission in 3 months | 0.656  0.196  0.319  11.571  0.651  0.418  1.164  7.389  0.891  0.632  6.128  0.979  0.839  0.999  4.544  1.164  0.867  2.168  1.078  1.96  1.234  3.82  1.006  1  0.403  1.459  2.727  1.499  1.934  12.865  0.975  0.98  3.182  0.845  0.949  1.006  0.453  0.969  1.534 | 0.266 – 1.622  0.051 – 0.754  0.051 – 1.99  1.307 – 102.451  0.253 – 1.673  0.17 – 1.462  0.316 – 4.295  0.75 –72.793  0.103 – 7.744  0.091 – 4.386  0.383 – 98.07  0.938 – 1.022  0.14 – 5.028  0.876 – 1.14  0.825 – 25.022  0.289 – 4.692  0.625 – 1.229  0.559 – 8.413  0.416 – 2.773  0.551 – 6.974  0.707 – 2.155  1.042 – 14.013  0.998 – 1.013  0.999 – 1  0.104 – 1.561  0.545 – 3.903  1.069 – 6.958  0.507 – 4.436  0.687 – 5.446  1.115 – 148.445  0.945 – 1.005  0.955 – 0.007  1.331 – 7.608  0.345 – 2.066  0.263 – 3.426  0.981 – 0.031  0.122 – 1.687  0.078 – 12.054  0.292 – 8.042 | 0.356  **0.018**  0.221  **0.028**  0.373  0.205  0.819  0.087  0.917  0.642  0.2  0.342  0.848  0.993  0.82  0.83  0.444  0.263  0.877  0.299  0.458  **0.043**  0.156  0.543  0.189  0.452  **0.036**  0.464  0.212  **0.041**  0.105  0.144  **0.009**  0.712  0.936  0.66  0.238  0.981  0.613 |

ASA, American Society of Anesthesiology; ASIA, American Spinal Injury Association; ATM, antithrombotic medication; BMI, Body mass index; CCI, Charlson comorbidity index; CI, confidence interval; CUP, cancer of unknown primary; DOACs, direct oral anticoagulants; GI, gastrointestinal; HACs, hospital-acquired conditions; ICU, intensive care unit; IQR, interquartile range; KPS, Karnofsky Performance Scale; LOS, Length of stay; OR, odds ratio; PMV, postoperative mechanical ventilation; PT, primary tumor; PSIs, patient safety indicators; RXT, radiotherapy; SM, spinal metastases; SSCs, spinal surgery-related complications; ST, systemic therapy; TTP, time to progression; VKAs, vitamin K antagonists.
